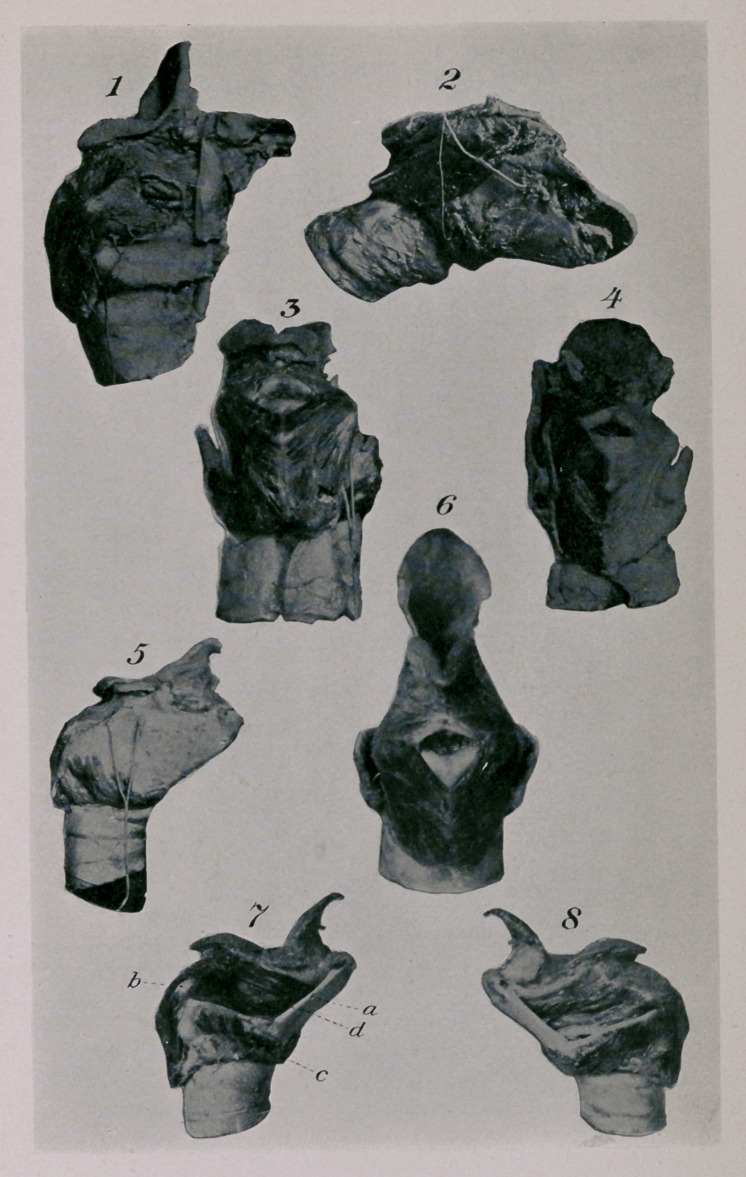# Hemiplegia Laryngis in the Horse (Roaring)1Paper read before the meeting of the Society for the Advancement of Veterinary Science, at Utrecht, September 22, 1900.

**Published:** 1901-05

**Authors:** M. W. J. P. Thomassen

**Affiliations:** Instructor at the Government Veterinary School at Utrecht


### THE JOURNAL

OF

### COMPARATIVE MEDICINE AND VETERINARY ARCHIVES.

VOL. XXII. *MAY, 1901.* No. 5.

### HEMIPLEGIA LARYNGIS IN THE HORSE (ROARING).[1]

BY PROF. M. W. J. P. THOMASSEN,

INSTRUCTOR AT THE GOVERNMENT VETERINARY SCHOOL AT UTRECHT.

*Translated from the Tijdschrift voor veeartsenijkunde,*

BY L. VAN ES, M.D., V.S.,

MEDICAL DEPARTMENT UNIVERSITY OF ALABAMA, MOBILE, ALA.

No disease or ailment of the horse is of more actual importance than roaring. To the most frequent—the nervous form—a hereditary character has been attributed for a long time, and therefore it is generally attempted to exclude from breeding animals affected with it.

The recognition of roaring--that is to say, the condition in which a horse makes an unusual noise after an exertion of shorter or longer duration (mostly by inspiration)—does not require much practical skill from the expert.

It is not so easy to determine whether the ailment is acute or chronic (that is, of a permanent nature), and, further, what is the cause of the roaring sound in question. Still this knowledge is, with a view to the possible heredity of the disease, of great importance.

How and by what the nervous form of the ailment is brought about remains, even for the most experienced expert, largely a conundrum. Our knowledge about its etiology and pathogenesis leaves yet much to be desired. Therefore, I occupied myself for some time with investigations, assisted by Mr. Kruijmel, for the purpose of penetrating the pathogenetic part of the question, and if possible of arriving at practical conclusions for the prophylaxis

[1] Paper read before the meeting of the Society for the Advancement of Veterinary Science, at Utrecht, September 22, 1900.

as well as for the curative treatment. Although we have not entirely succeeded, I think I am already able to communicate something of the results of the first part of this investigation. At the same time our work may serve to refresh the memory of many among you in relation to facts already known. By the demonstration of normal as well as pathological preparations which I have collected I will endeavor to illustrate my work.

I will limit myself, for the time being, to the consideration of facts, giving the first part of my investigation with the hope to add at a future meeting something about etiology and therapy. For the confirmation of what at present are surmises or hypotheses many experiments are yet to be made.

In speaking of stridor laryngis one must accept that a narrowing (stenosis) of this part of the air passages exists.

The sound, which can be caused by narrowing of the foremost air passages, differs so much from the laryngeal stenosis sound that the nasal dyspnœa may remain entirely out of consideration. Moreover, in this form certain other typical symptoms never fail, which make a tolerably correct diagnosis possible, and in this case the word roaring is usually not employed.

Before entering more closely into the causes of laryngeal stenosis it is well to deal shortly with the structure and normal function of this important organ, partly in coarse outlines, partly, however, in more minute details, in so far as it must serve for the illustration of the pathological part.

*Anatomy.* The larynx forms the entrance of the trachea, adjustable by muscular action, whereby its lumen becomes modified according to the force of the stream of inspired air. The larynx is the voice-producing organ, wherein by man and animals different sounds are produced by means of tension and relaxation of the vocal cords. It serves also as a guard for the deeper air passages, as by the least irritation of the mucous membrane the glottis closes itself, whereby the entrance of foreign bodies and of irritating gases is prevented as much as possible. In the larynx, furthermore, originates the natural protection reflex—the cough.

*Cartilages.* You know that the framework of the larynx consists of one pair and three single cartilages.[1] Of the latter, the epiglottis has for our subject the least significance. It serves to close the larynx, but less, however, than the arytenoid cartilages, which form the most motile part. The external shape of the

[1] In the original Prof. Thomassen treats the thyroid cartilage as a pair cartilage; but for convenience of our custom we have adopted the ordinary view of it as a single body.—Ed.

larynx is principally outlined by the thyroid cartilages. The cricoid cartilage, along with the arytenoids and the vocal cords, contributes the most toward the modifications of the calibre of the glottis. The glottis is the narrowest aperture of the larynx. It forms an elongated triangle, of which the apex points forward toward the anterior angle of the thyroid cartilage, while the base is situated between the arytenoids.

The narrowest space between the vocal cords is at rest from 3 to 5 mm., and is called the *glottis vocalis*. The larger almost ovoid space between the arytenoid cartilages has in its widest parts a diameter of 1½ to 2 cm., and is called *glottis respiratoria*.

The vocal cords contain much elastic tissue, by which they are permanently tense. They partly enclose the thyro-arytenoideus inferior muscle, to which they owe their volume. Between both the thyro-arytenoid muscles the ventricle (*ventriculus Morgagni*) is situated.

A phonation, cadaver, and inspiration posture of the glottis is spoken off. During phonation the vocal cords approach one another over their whole length to such an extent that only a narrow fissure between the edges remains open.

Elevation and close adjustment of the arytenoid cartilages against the thyroid gives rise to dilatation, which is especially marked during forced inspiration. The vocal cords follow this movement. The change in the position of the glottis is brought about by muscular action.

*Muscles.* The laryngeal muscles are divided in dilators (abductors) and constrictors (adductors). In veterinary science the arytenoideus transversus and formerly also the crico-arytenoideus lateralis are regarded as dilators besides the crico-arytenoid and crico-thyroid muscles. The principal—we could almost say the only—dilator is the crico-arytenoideus posticus. This muscle elevates the arytenoid cartilage outside the larynx, stretches the vocal cords, and closes the entrance of the ventricle. Of the crico-thyroid it can only be said that it widens the glottis. By tilting of the bezel or ringplate the arytenoids are lifted upward and outward from the thyroid cartilages. All the other muscles are constrictors. We have been able to prove this by direct electrical irritation of the muscles by means of a Du Bois-Reymond apparatus. The irritation of the arytenoideus transversus, unilateral as well as bilateral, causes the larynx to constrict. The position of this muscle in front of the crico-arytenoid articulation proves sufficiently that it is only able to pull the arytenoid cartilages together. Moreover, the continuity of a large part of its fibres, with those of the thyro-arytenoideus superior, permits only this action. According to other anatomists, it acts as a constrictor or dilator, dependent upon its contraction with constrictors or dilators.

I saw, however, also, constriction by electrical stimulation of the arytenoideus[1] transversus after this one had been separated from the thyro-arytenoideus superior. Stimulation of both the arytenoidi transversi causes the arytenoid cartilages and the vocal cords to approach one another. On simultaneous irritation of the arytenoideus posticus and transversus less marked dilatation followed than when the first was stimulated by itself. The crico-arytenoideus lateralis and the thyro-arytenoidi superior et inferior are all constrictors. The tensors of the vocal cords are first the thyro-arytenoideus inferior and then the crico-thyroideus.

*Nerves.* The muscles of the larynx, with the exception of the crico-thyroideus, receive all branches of the inferior laryngeal (recurrent) nerve. As is plainly visible on this preparation, the recurrent gives off first one, sometimes two branches to the posticus muscle. Somewhat higher a smaller branch passes obliquely backward and upward under the muscle mentioned to divide itself afterward in the half of the transversus muscle of the same side. The small trunk, continuing, folds itself upward, runs between the thyroid and cricoid cartilages, and first gives off two branches to the lateralis and then branches to both the thyro-arytenoid muscles. In regard to the crico-thyroid muscle most of the veterinary authors treating of roaring, as Günther, Möller, Cadeac, Fröhner, etc., maintain that this muscle of the horse receives its innervation by a small branch of the first cervical nerve. The writers first mentioned even dispute with one another the priority of the discovery. In man this muscle receives a branch from the superior laryngeal nerve; in the dog the existence of a median laryngeal nerve has been conceded, which, along with the superior, supplies the innervation of this muscle. The freak of nature in the horse, described above, must cause anyone to wonder.

We often tried on the cadaver to convince ourselves of the truth of this fact, but searched in vain for a nerve branch to the muscle in question, derived from the first cervical nerve. From this preparation it will appear to you that we are confronted by an anatomical error. (Fig. 2.)

[1] In the future we will speak simply of the posticus transversus, lateralis, superior, inferior, and crico-thyroideus for the sake of brevity.

Following the delicate nerve bundle upward, which plainly enters the posterior part of the crico-thyroid muscle, we see that it runs over the crico-pharyngeal muscle and further obliquely forward and upward for a distance of about 10 cm. and there connects with the ramus pharyngeus, about 4 cm. under the spot, where it has left the vagus plexus in the neighborhood of the ganglion supremum. It appears to us that this pharyngeal branch itself originated partly from the sympathetic.

In two other horses I found the course somewhat different. The nerve branch came from the plexus formed by the vagus, just below the place where the superior laryngeal leaves it. The superior laryngeal, derived from the vagus, enters, in the horse, an opening situated between the hyoid bone and the posterior edge of the thyroid cartilage, to distribute itself in the mucosa and the pharyngeal muscles, at the same time having formed anastomoses with the recurrent. Sometimes an important anatomical deviation in the course of this nerve can be observed, which will be mentioned later. Passing the undecided question whether the motor fibres of the laryngeal nerves are derived from the spinal accessory or vagus, it is at any rate certain that also in the horse all nerves destined for the larynx, motor as well as sensory, after union of the above-mentioned nerves at their exit through the jugular foramen, run along the course of the vagus, from which, first, the superior laryngeal, then the branch for the crico-thyroid muscle, and only after reaching the thorax the inferior laryngeal is given off. The first cervical nerve thus does not supply any of the laryngeal muscles of the horse. We consider these anatomical details to be sufficient for the time being. We will have occasion to enter more closely into the physiological part, where this may prove necessary for the illustration of pathological characteristics.

*Pathology of the Larynx.* At the beginning it was stated that stridor laryngis points toward stenosis laryngis. How can this constriction of the larynx originate? It is generally accepted that in the horse in at least 95 per cent. of the cases unilateral paralysis of the larynx is the existing cause of the ailment.

*Deformity Stenosis.* The other 5 per cent. are due to tumors, granulations, malformations, etc., in the larynx, sometimes in the trachea. I have the opportunity to show you some specimens of the last-mentioned lesions:

1. The trachea of a pony, fully twenty years old, which, after some exertion, produced during expiration a wheezing sound. As you see, there are found in the trachea, from the bifurcation as far as the cricoid cartilage, three rows of closely connected nodules the size of a pea and larger. By closer examination these tumors proved to be enchondromata.

2. Larynx of a roarer, with a polypus under the right vocal cord and two smaller ones between the arytenoid cartilages. In 1885 I saw another case of roaring, by which the stenosis was caused by a polypoid growth the size of a nut. This also was attached to the right vocal cord.

3. Larynx with vegetations on the superior edge and on the internal surface of the right arytenoid cartilage, giving rise to considerable narrowing of the entrance and stridor.

4. Recently I found in a stallion a tumor situated on the posterior surface of the posticus, which by degeneration at the muscle caused a severe degree of roaring.

*Nervous Form.* In 95 per cent. of the cases the stenosis is the result of a hemiparesis or hemiplegia usually of the left half, whereby either all muscles except the crico-thyroid are more or less evenly degenerated, or only a few, among which the posticus commonly occupies the first place. Sometimes the atrophy of the adductors is very conspicuous. The total, even the partial paralysis of this dilator of the larynx, is sufficient to produce a stenosis sound.

It is fairly well decided that in man and in animals this unilateral muscle degeneration, with atrophy of some or all of the muscles, is not based upon a myopathic affection. The ailment is of neuropathic origin. In view of the fact that human medicine is far ahead of veterinary medicine, especially on the subject of nervous diseases, I consider it desirable to consult it for a moment on this important ailment, notwithstanding that laryngeal paralysis occurs much more frequently in the horse than in man. According to its origin we distinguish in human medicine the cerebral (cortical), the bulbar,[1] and the peripheral form of laryngeal paralysis.

(*a*) The cerebral (cortical form). Semon[2] believes that by lesions of the cortex, as well as of the tracts running from there into the bulbus, paralysis of the larynx can be caused, which is then limited to a functional disturbance of the constrictors, the muscles, which are, under the influence of the psychomotor centre, involved. A voluntary function—phonation—is caused by it.

[1] Bulbus is a name commonly used by Continental writers to designate the medulla oblongata.

[2] P. Heymann. Kehlkopf und Luftrhöre. Part II. Nervous Disturbances Worked Out by Semon. Wien., 1898.

Respiration largely takes place automatically, but may also be brought under the influence of the will. Semon saw this paralysis always occur bilaterally. Other writers, however, mention unilateral paralysis, caused as the result of brain lesions. It is plain that the laryngeal paralysis by unilateral affection is accompanied by other symptoms brought about as a consequence of destruction by hemorrhage, tumors, etc. In man we recognize functional and organic paralysis of the larynx. In the first the anatomical changes escape observation.

In the functional form the constrictors only are, almost without exception, involved, so that the psychical or phonation function of the larynx exclusively is disturbed. The physical or respiratory function remains intact. The origin of the tracts of the first has its seat in the cortex of the cerebrum; of the second in the medulla, although, according to others (Russell), special centres for the respiration are also to be found in the cortex. In the recurrent we find these tracts in the form of two sharply distinct nerve bundles in a common sheath (will be alluded to later). Bilateral functional disturbances of the larynx of a central origin nearly always occur with other psychical disturbances. Among others hysteria, after-emotions, shock, etc., and, as already stated, limit themselves to phonatic ones. There cannot be any more question of a functional paralysis of the posticus than of a totally functional paralysis of the recurrent. Semon only knows a symmetrical and not a unilateral paralysis of the adductor group. This decision is based upon clinical but not on experimental observations. He does not deny the possibility of cases, as reported by Donaldson and P. Heymann, whereby paralysis of the crico-arytenoideus lateralis occurred They may be the result of purely local processes, whereby, just as well, paralysis of other constrictors may be produced.

Can we also make this distinction in the horse? In other words, has there been observed in this animal species a distinct degeneration of the constrictors, so that in the animal disturbances of phonation only and not of the respirators are noticed? For a part we are able to answer this question in the affirmative and well in this sense: that a distinct atrophy of the constrictors, but unilateral and left-sided, is seen now and then, the cause of which, however, must be looked for probably in the periphery. We will return to this later on, and will show you a larynx on which the lesions are plainly to be seen.

(*b*) Bulbar form. There may occur as a result of bulbar lesions a distinct paralysis of the dilators (posticus) or a total recurrent paralysis (dilators and constrictors), unilateral or bilateral, isolated or along with other cerebral paralyses. In man this laryngeal paralysis is often seen in the course of tabes dorsalis, but also as a consequence of hemorrhages, tumors, and syphilitic affections of this part of the central nervous system. Although its possibility is not excluded, the occurrence of this form in the domestic animals, and especially in the horse, belongs to the greater rarities. In the so-called lumbar prurigo of sheep there occurs a similar disturbance of phonation, perhaps also of respiration ; it may be, also, in the "throat disease" (bulbar paralysis) of the horse, which has frequently been observed in Flanders.

(*c*) Peripheral form. In veterinary pathology this deserves the greatest interest. In the very large majority of the cases the nervous form of roaring in the horse is an ailment of a purely peripheral nature, and, considering the innervation of the laryngeal muscles, there can only be considered disease of the recurrent nerve or at the vagus.

Before dealing further with the clinical paralysis of the recurrent, it is useful to become acquainted with the consequences of nerve resection and nerve stimulation of the superior as well as of the inferior laryngeal nerve, and to watch the results in the larynx hereby brought about.

*The Experimental Paralysis of the Recurrent Nerve.* Galenus is credited as the discoverer of the recurrent nerves. He severed them at first in swine, and only points to the loss of voice (aphonia) as the result of the operation. Since the beginning of the century the resection of the recurrent has been frequently practised in the dog.

Le Gallois[1] has observed that young dogs during the first days after birth rapidly succumb to the consequences of a double resection. At the age of three months they survive the operation, and later on the aphonia is the only damage they experience. When the animals exert themselves an abnormal inspiration sound is heard.

In the main the same results have been obtained by later experimenters. The vocal cords remain permanently in the cadaver position after having been temporarily in the median posture for a couple of days.

We will not expatiate on the question why the vocal cords after resection of both the recurrents temporarily assume the median in

[1] Expérience sur le principe de la vie. Paris, 1812.

place of the cadaver position. This for our object is of little significance. Only let it be mentioned that some (Longet, Le Gallois) regard this as the result of the inspiratory rarefication of air below the glottis, by which is caused the approaching of the vocal cords, at least in young animals. The same thing is seen in the excised larynx by the suction with a syringe in the trachea. At a later age, when the anterior processes of the arytenoid cartilage are fully developed, only half of the glottis is limited by the true vocal cords. The cartilaginous portion, then, remains free for the respiration. Semon proved that after opening the trachea the median position suddenly comes to an end. This was confirmed by Onodi. Klemperer says the dyspnœa aggravates the stenosis.

Wagner and Grossmann, on the contrary, consider the median position a result of the action of the crico-thyroid. They assert that by placing this muscle out of function the glottis rapidly would change from the median to the cadaver position. By section of the nerves a temporary complete adduction, for example, of a half an hour may result as a consequence of nervous irritation.

Cats, guinea-pigs, and rabbits also have frequently served for this experiment. In cats the consequences of a bilateral operation are more serious than in the dog. Even full-grown animals may succumb immediately after the operation. The stenosis sound soon diminishes if they survive the first perturbation. Sometimes the respiration is perfectly quiet after a few hours. The vocal cords also here change from the median to the cadaver position.[1] Rabbits tolerate the consequences of the operation still better than dogs. Even during the first days of life no suffocation occurs. Excitement may produce stridor during inspiration and expiration. The guinea-pig is in this respect about equal with the dog.

In the bovine a unilateral resection of the recurrent does not lead to roaring. Of this I was able once to convince myself. In general, the rule prevails that the position of the glottis in the cadaver is a good indication of its width after nerve resection.

By this can be explained the different results obtained in different species of animals—even in animals of the same species. The results observed in the horse are for our subject of more significance, and deserve, therefore, a more detailed consideration.

About 1821, Dupuy already had severed the nerve or placed it out of function by pressure, and in this manner caused roaring

[1] For experiments on dogs and cats vide: Zur Stimm Candstellung nach Recurrensdurchschneidung, etc. By Dr. H. Burger. Berlin, 1899. Archive für Laryngologie, 9 Band, 2 Heft.

and showed atrophy of the laryngeal muscles after death. In England, Youatt experimented in 1838 by ligation, and afterward by resection of the recurrent nerve.

Günther (father), who occupied himself for years seriously with the study of roaring, reports three resections in this animal in his exhaustive treatise.[1]

1. In the first case it concerned an old mare which, after resection of the right nerve, roared more or less during locomotion only.

2. A horse of medium age became so distressed after the severing of the two recurrents that in order to prevent asphyxia tracheotomy was urgently necessary.

3. In a horse, aged twenty-six years, after resection of the two recurrents little was to be noticed when at rest. When trotting stridor appeared but without danger of asphyxia.

Other authors casually mention mostly unilateral neurectomy, and limit themselves to the report of the symptoms of stridor during movement. Longet[2] points to the difference of the results of recurrent resection in old and in young animals, for which he already had an explanation. I had occasion to perform nerve resection in horses of different ages, but always unilateral. Animals above twelve years old became roarers only in a very slight degree, so that during trotting movement of more than five minutes only an insignificant stenosis sound was observed. Also, in young horses the stridor was never so intense as it is heard in spontaneous unilateral recurrent paralysis.

1. After resection of a part of the right recurrent nerve in a mare, aged nearly five years, roaring could be heard at a distance during trotting movement immediately after the operation. Two days after the operation the sound was heard when she was led in a trot or gallop only in the immediate vicinity of the animal.

As soon as the animal came to a standstill nothing more could be heard. Before the section the nerve had been electrically irritated, which will be spoken of hereafter.

2. A horse, aged about ten years, still roared fully three months after the resection of the right recurrent nerve, but in a slight degree. The sound was less intense than in the first days after the operation. After opening the larynx a local examination, however, revealed that the right arytenoid cartilage, almost immovably, remained in the cadaver position Here you see the larynx of the horse in question with a large piece of the recurrent and the

[1] Zeitschrift für die gesammte Thierheilkunde und Viehzucht. Von Nebel und Vix, 1834.
[2] Traité de Physiologie, 1861, T. i.

vagus. Immediately after the section of the nerve its peripheral portion was stitched to the vagus by means of catgut. The nerves are grown together tolerably well.

As appears from these preparations I had been able to convince myself of this in a couple of cases as early at 1897. Although the horse was destroyed fully three months after the operation, atrophy of abductors as well as adductors is hardly observable. The muscles of the right half, however, have largely lost their cross striped appearance. (Fig. 3.) I will not venture to decide if here total recovery of the nerve course was to be expected. This is to be learned from further experimentation. At any rate, it is sure that the stridor was considerably less about three weeks after the operation than during the first days.

3 and 4. In a couple of other cases the atrophy of the laryngeal muscles after resection of the nerve is much more marked, although the animals were destroyed as early as two months after the operation. That both horses roared little explains itself by their age, whereby more rigidity of the soft parts of the larynx, consequently less marked median displacement of the arytenoid cartilage. I can show you the larynx of both animals. First, the larynx of a pony, aged twenty years, and, furthermore, one of a horse, aged about fourteen years, in which about two months before death the left recurrent had been severed.

The atrophy of the dilators and constrictors of the left half is very plain. The muscles are also paler than those of the other side. By microscopical examination the usual degeneration phenomena are found. Notwithstanding those conspicuous changes roaring existed only in a slight degree.

Other horses on which recurrent resection was performed lived only a short time after the operation, so that anatomical changes were yet absent. This holds good of the preparation with branched laryngeus superior (Fig. 4) belonging to a five-year-old horse which was operated on three days before death. Of this animal mention has been made.

(To be continued.)

### DESCRIPTION OF PLATE.

FIG. 1.—Nervus recurrens with branches in the laryngeal muscles.

FIG. 2.—Vagus nerve with plexus, below which the ganglion supremum of the nervus sympathicus ramus pharyngeus, from which originates the small nerve branch which goes to the crico-thyroideus muscle. Posterior, the superior laryngeal nerve, which penetrates the larynx near the upper corner of the thyroid cartilage.

FIG. 3.—Larynx of a ten-year-old horse on which resections of the recurrent nerve were practised three months before death. The right posticus muscle is somewhat pale and atrophic. The peripheral portions of the recurrent nerve were immediately attached to the vagus, and at the autopsy were found to be united with the latter.

FIG. 4.—Superior laryngeal nerve, which divides in two.

FIG. 5.—Larynx of a two-year-old horse, with marked atrophy of the right posticus and transversus.

FIG. 6.—Larynx of a three-year-old stallion, with atrophy of the left half of the transversus and a nearly normal left posticus.

FIG. 7.—The same, with marked atrophy of the left constrictors, crico-arytenoideus lateralis, and thyro-arytenoideus inferior. The thyro-arytenoideus superior is less atrophic than both the other adductors.

FIG. 8.—The same, seen from the right, where the three constrictors are absolutely normal.

## Figures and Tables

**Figure f1:**